# TBK1 is paradoxical in tumor development: a focus on the pathway mediating IFN-I expression

**DOI:** 10.3389/fimmu.2024.1433321

**Published:** 2024-08-05

**Authors:** Banglu Wang, Fan Zhang, Xiaoyu Wu, Mei Ji

**Affiliations:** Department of Oncology, The Third Affiliated Hospital of Soochow University, Changzhou, China

**Keywords:** TBK1, IFN-I, immunotherapy, TBK1 inhibitor, innate immunity

## Abstract

TANK-binding kinase 1 (TBK1) is a member of the IKK family and plays a crucial role in the activation of non-canonical NF-κB signaling and type I interferon responses. The aberrant activation of TBK1 contributes to the proliferation and survival of various types of tumor cells, particularly in specific mutational or tumorous contexts. Inhibitors targeting TBK1 are under development and application in both *in vivo* and *in vitro* settings, yet their clinical efficacy remains limited. Numerous literatures have shown that TBK1 can exhibit both tumor promoting and tumor inhibiting effects. TBK1 acts as a pivotal node within the innate immune pathway, mediating anti-tumor immunity through the activation of innate immune responses. Facilitating interferon-I (IFN-I) production represents a critical mechanism through which TBK1 bridges these processes. IFN has been shown to exert both beneficial and detrimental effects on tumor progression. Hence, the paradoxical role of TBK1 in tumor development may necessitate acknowledgment in light of its downstream IFN-I signaling cascade. In this paper, we review the signaling pathways mediated by TBK1 in various tumor contexts and summarize the dual roles of TBK1 and the TBK1-IFN pathways in both promoting and inhibiting tumor progression. Additionally, we highlight the significance of the TBK1-IFN pathway in clinical therapy, particularly in the context of immune response. We anticipate further advancements in the development of TBK1 inhibitors as part of novel cancer treatment strategies.

## 1 Introduction

The non-classical member of the IKK family, TBK1, derives its distinction from notable structural disparities while maintaining akin biological functions to its classical IKK counterpart. Although both kinases exhibit a preference for phosphorylating similar motifs and share substrates, their disparate regulation and involvement in distinct protein complexes imply potential discrepancies in signaling (1). In the nascent stages of innate immune response research, TBK1’s paramount function is often attributed to its mediation of IFN-I production via the NF-κB and IRF pathways (2). Nevertheless, TBK1-mediated IFN-I, autoantibodies, and chemokines also contribute to the onset of autoimmune diseases in humans (3). Further research has revealed TBK1’s involvement in various aspects of tumorigenesis, including supporting tumor angiogenesis (4), mediating tumor-related autophagy (5), regulating cell cycle and mitosis (6, 7), and inducing epithelial-mesenchymal transition (EMT) (8).

IFN-Is, comprising 13 isoforms such as IFN-α, IFN-β, IFN-ω, and IFN-ϵ, play pivotal roles in both antiviral and antitumor immunity. IFN-I receptor, collectively IFNAR, plays an important role in antiviral defense and is universally expressed on all nucleated cells. This widespread presence implies that virtually every cell has the potential to respond to IFN-Is. However, the sensitivity to IFN-Is varies among different cell types, leading to diverse effects depending on the cellular context (9). Nonetheless, prolonged IFN-I signaling can lead to chronic inflammation and immune dysfunction. Similarly, in cancer, IFN-I can elicit antitumor immune responses but may also foster tumor progression through chronic inflammation (9). Furthermore, apart from STAT1/STAT2 signaling, IFN-Is can activate additional pathways such as STAT3–6 and STAT-independent pathways like JNK, ERK, p38 MAPK, and mTOR. These pathways manifest in diverse and sometimes conflicting immunological effects mediated by IFN-I signaling (10). Phagocytes internalize tumor fragment DNA to stimulate the secretion of IFN-I and the exogenous recognition by CD8+ T cells, thereby engaging in the tumor immune response (11). However, endogenous IFNα promotes the expression of PD-1/L1 in the tumor microenvironment, mediating immunosuppression (12). The efficacy of IFN-Is is intricately influenced by various factors, encompassing isoform variation, as well as timing, cell type, and the surrounding inflammatory milieu (10).

TBK1 has been identified through genome-wide screening as a site of carcinogenesis with upregulated expression in numerous tumors, correlating with poor prognosis (13), rendering it an appealing target for robust cancer suppression. However, TBK1 inhibitors have not yielded optimal early clinical outcomes. Momelotinib, the sole TBK1 inhibitor undergoing clinical trials in oncology, failed to confer desired anti-tumor benefits through the intended therapeutic molecular mechanism (14). Scientists early recognized TBK1 as a lethal partner of KRAS (15). However, targeting TBK1 in tumor cell lines harboring KRAS mutations failed to significantly impede tumor growth (16). Apart from limitations in the potency and specificity of the drug itself, a primary reason is the lack of comprehensive understanding of tumor types and the inherent heterogeneity of tumor subtypes dependent on TBK1. Regulation of IFN-I expression is a significant mechanism underlying TBK1’s role in tumor development. IFN-I exerts a dual role in tumor immunity, both inhibiting tumor growth and promoting tumor progression. By synthesizing the activation and mechanistic pathways of TBK1 across diverse tumor backgrounds, this study deeply delved into the anti-tumor and pro-tumor effects of TBK1 regulation on IFN-Is, along with the significance of TBK1-IFN-IS in immunotherapy, offering novel insights and strategies for the more effective utilization of TBK1 inhibitors in clinical settings.

## 2 Tumor background of TBK1 activation or inhibition

The upstream and downstream signal transduction network of TBK1 is intricate, allowing for selective activation of downstream targets in specific diseases and pathological states while avoiding excessive pathway activation through selective splicing (17). Additionally, the subcellular localization of TBK1 is regulated by the selective binding of specific adaptor proteins. These adaptor proteins guide TBK1 into particular cellular compartments and control its activity and substrate specificity (18). Notably, TBKBP1, a crucial adaptor protein for TBK1, mediates the MTORC1-activated growth factor signaling pathway, which is essential for tumor growth. This finding highlights an additional significant role of TBK1 beyond its induction of type I interferon production. Furthermore, this study discovered that TBK1 mediates tumor T cell depletion and glycolysis, thereby contributing to immunosuppression (19). Moreover, previous studies have demonstrated TBK1’s involvement in immune tolerance and adaptive immune regulation (20). It has been observed that TBK1 is upregulated in various tumors, with its expression inversely correlated with immune cells other than CD4 T cells in the tumor microenvironment (21). For instance, TBK1 phosphorylates AGO2, which functions with double-stranded miRNA, to generate carcinogenic miRISC via the S417 site, a process related to the resistance of gefitinib targeted therapy in non-small cell lung cancer (22). In-depth research into this mechanism has shown that TBK1 inhibitors can provide a solution to gefitinib resistance, thereby expanding the scope of clinical applications.

**Figure 1:**
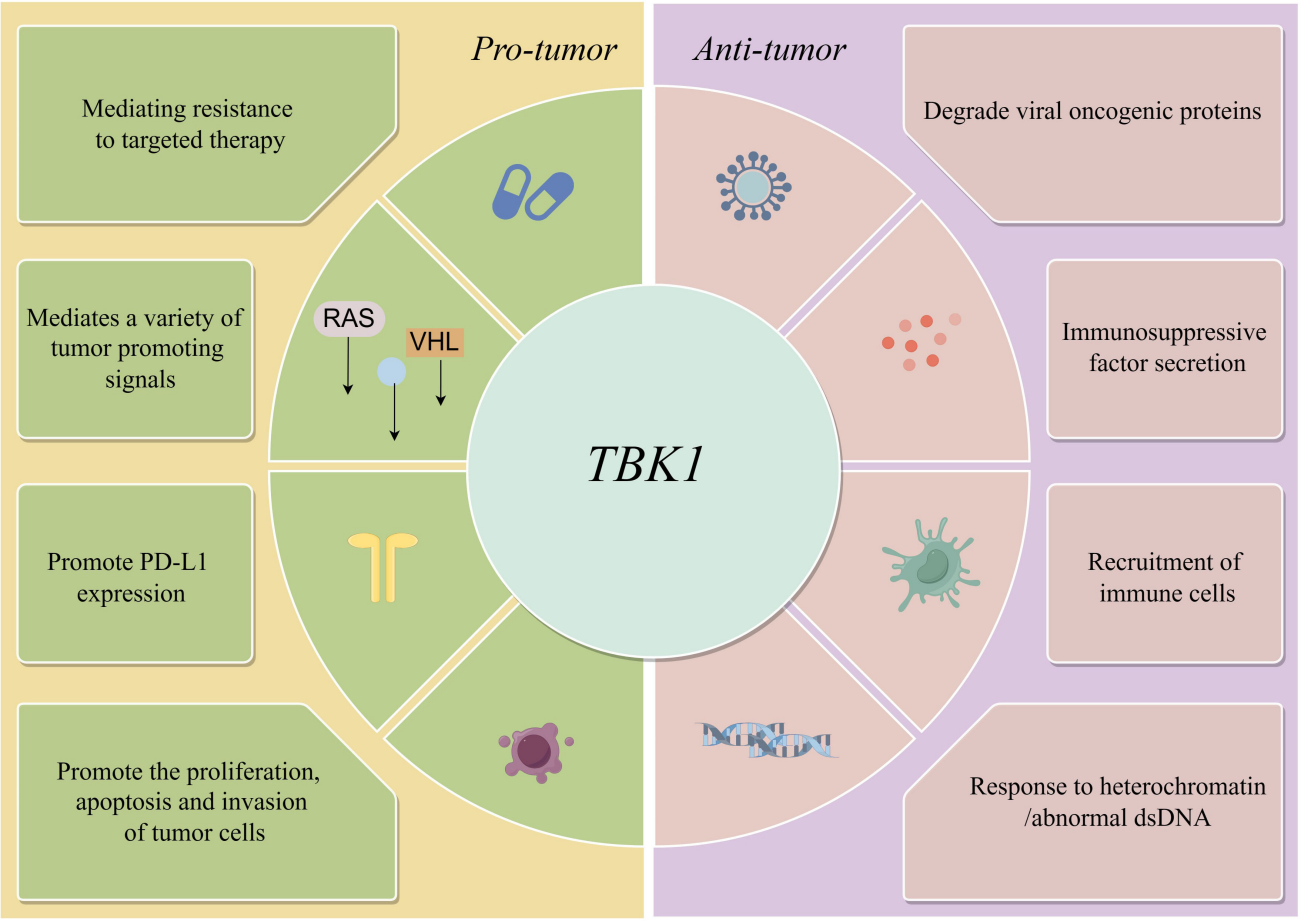
Anti-tumor and pro-tumor pathways of TBK1.

However, in some cases, TBK1 does not always function as an active pro-tumor factor. Adaptive resistance arising from specific mutations is closely associated with the TBK1-mediated inhibition of innate immune pathways, particularly when TBK1 acts as a key downstream node in the cGAS-STING signaling pathway. For instance, MET-amplified drug-resistant tumor cells diminish immunogenicity by suppressing stimulator of interferon genes (STING)-dependent TBK1-IFN signaling through CD73 (23). Additionally, Mouse models have demonstrated that the effective activation of CD8 T cells within tumors relies on the activation of dendritic cells via the cGAS-STING pathway (24). The role of STING agonists in tumor suppression is well-recognized, and their combination with immunotherapy holds significant potential (25). Nevertheless, the use of TBK1 inhibitors requires careful consideration of tumor-specific signaling. In the case of intestinal tumors, the application of TBK1 inhibitors may need more careful evaluation, especially in the scope of STING agonists. It has been shown that TBK1 deficiency in intestinal epithelial cells enhances macrophage expression of IL1β, thereby promoting the differentiation of CD4 T cells into Th17 cells and exacerbating the inflammatory response. In this context, TBK1 assumes a distinct pro-pathological role compared to other tumors (26). Hence, comprehending the role and mechanism of TBK1 in diverse tumor backgrounds holds significant value in maximizing the efficacy of TBK1 inhibitors (**Figure 1** **)**(**Table 1**).

**Table 1:** Physiological role of TBK1 in different tumor backgrounds.

Cancer	characteristics	Tbk1-mediated signaling pathway	Activate/Restrain	Physiological effect	Reference
NSCLC	MET amplification	STING-TBK1-IRF3-IFN I	Restrain	Increase immunogenicity	(23)
	EGFR inhibition	RIGI-TRIM32-TBK1-IRF3-IFN I	Activate	Mediates resistance to EGFR inhibition	(27)
	A20-deficient	TBK1-IFN I-STAT1-IFNγ-PD-L1	Activate	Promote immune escape	(28)
	WEE1 inhibition	STING-TBK1-IRF3-IFN I-CXCL10/CCL5	Activate	Promote CD8 cytotoxic T cell infiltration	(29)
	KRAS	TBK1-PLK1	Activate	Regulate mitosis	(6)
	/	TBK1-NF-κB/KRAS-RALB-TBK1/IKKϵ-CCL5、IL6-JAK/STAT3	Activate	Autophagy addiction	(30)
	Mesenchymal, Ras-mutant	TBK1-AKT/mTOR	Activate	Support tumor survival	(31)
	Radiotherapy	TBK1-GSK-3βand ZEB1/TBK1-AKT-ERK	Activate	Regulates radiation-induced EMT	(32)
	KRAS-LKB1 mutant	mt-dsDNA-STING-TBK1-IRF3/STAT1	Restrain	In response to mitochondrial dysfunction	(33)
breast cancer	DDRD	cGAS-STING-TBK1-IRF3	Activate	activated expression of PD-L1	(34)
	DDRD	TBK1-FOXO3A-ERα	Activate	EMT	(35)
	PKCλ/ι deficiency	ULK2-TBK1-IFN	Activate	Recruitment of CD8+ T cells	(36)
	PTEN null TNBC	Rab7-STING-TBK1-IRF3	Activate	Mediates the production of chemokines	(37)
Colorectal cancer	frameshift mutations of RIG-I	/	Activate	non-specific inflammatory response	(38)
PDA	KRAS	Axl-TBK1	Activate	epithelial-mesenchymal transition	(39)
kidney cancer	VHL loss	/	Activate	promote renal tumorigenesis	(40)
HNSCC	Up-regulated AKLBH5	RIG-I -IKKϵ/TBK1-IRF3-IFNα	Restrain	Promoting immune-killing cell infiltration	(41)
/	Glioblastoma Cancer Stem Cells	TLR4-TBK1-RBBP5	Restrain	Evade Innate Immune Suppression of Self-Renewal	(42)
ovarian cancer	Up-regulated USP35	STING-TBK1-IRF3-IFNI	Restrain	Regulation of cisplatin sensitivity	(43)
	BRCA1-deficient	STING-pTBK1-IFN	Activate	leads to a cell-autonomous inflammatory state	(44)
cervical cancer	/	STING-TBK1-NF-kB	Activate	upregulating PD-L1	(45)
EC	MSI	TRIM14-TBK1-IRF3-IFN-β	Activate	promotes CD8T cell exhaustion	(46)
prostate cancer	/	IKK-ε/TBK1-NF-κB	Activate	EMT	(47)
acoustic neuromas	mutant NF2	cGAS-STING-TBK1-IRF3	Restrain	abolishes STING-initiated antitumor immunity	(48)
AML		IKBKE/TBK1-YB-1-MYC	Activate	drive MYC expression	(49)
melanoma	mutant NRAS	/	Activate	Promote tumor cell invasion	(50)
/	expression αvβ3	αvβ3-KRAS-RalB-TBK1/NFκB	Activate	promote cancer stemness and drug resistance	(51)

DDRD (DNA damage response–deficient), PDA (Pancreatic ductal adenocarcinoma), VHL (von Hippel-Lindau), HNSCC (head and neck squamous cell carcinoma), MSI EC (microsatellite instable endometrial cancer), AML(acute myeloid leukemia).

## 3 Understanding and application of the dual role of TBK1-IFN pathway in tumor development

TBK1, ubiquitously expressed across tissues, serves as a central node in multiple IFN generation pathways. It responds to abnormal DNA and RNA *in vivo*, receiving signals from RNA and DNA sensors such as MDA5, RIG-I, cGAS, and DAI (52). These signals can originate from both autologous and non-autologous DNA and RNA. Upon activation, TBK1 is assembled by three mutually exclusive scaffold proteins of TANK (53), NAP1 (54), and SINTBAD (55). The activation of the IFN pathway was most closely related to the TANK located in the perinuclear region. This assembly mediates the phosphorylation and nuclear translocation of IRF3/7 (56), which then binds to ISREs in target gene promoters (e.g., IFNB and RANTES) (57), thereby initiating the IFN signaling pathway and regulating gene expression. This process involves recruiting co-activators p300 and CBP, and cooperating with NF-κB (58).

**Figure 2:**
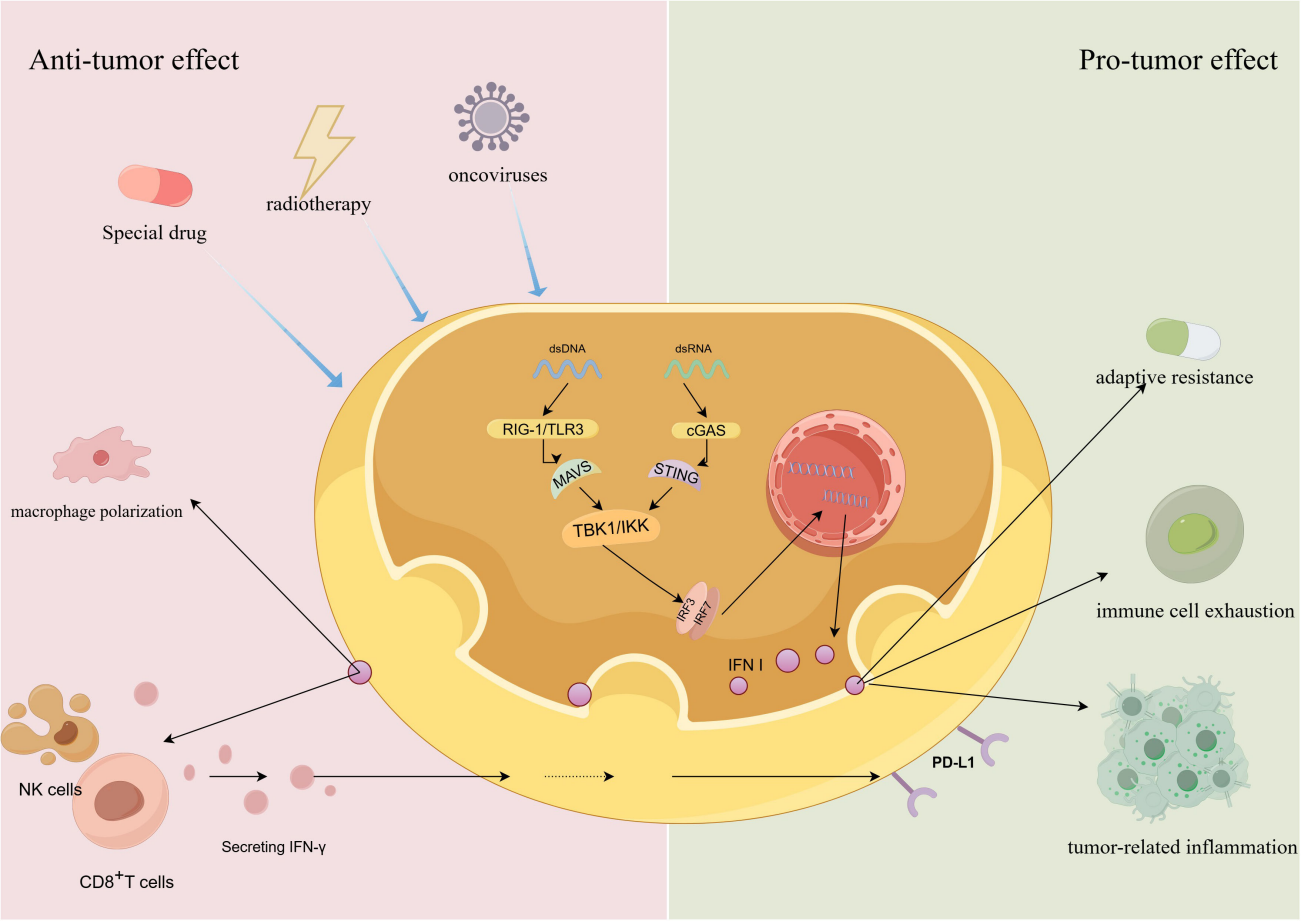
The dual role of TBK1-IFN pathway in tumor development.

Elucidating the dual effects of TBK1 on tumors is challenging due to the intricate upstream signaling pathways and the spatial and conditional contexts of its activation. This complexity hinders the development and application of TBK1 inhibitors. As a critical downstream pathway of TBK1, the dual role of IFNs in tumor progression has garnered attention, potentially offering key insights into the bifunctional nature of TBK1(**Figure 2**).

### 3.1 Antitumor effect

Cancers often evade immune detection by suppressing STING-IFN signaling. Kaposi’s sarcoma-associated herpesvirus, associated with various tumor incidences, inhibits the interaction between STING and TBK1 through vIRF1, thereby blocking IFN-β generation and promoting tumor occurrence and viral spread within the population (59). Conversely, vanillic acid (VA) activates the STING/TBK1/IRF3 pathway, promoting the production of type I IFN. This activation induces IFNβ production via STING, which is crucial for polarizing macrophages into an anti-tumor phenotype. Furthermore, IFN, combined with the IL-6R/JAK signaling pathway, enhances macrophage phagocytosis and induce apoptosis, contributing significantly to its anti-tumor effects (60). Moreover Macrophages can regulate RIG-I-TBK1-IRF3-mediated IFN responses via TIPE2 during antiviral periods (61). In the context of HPV infections, IFNs can overcome viral immune evasion strategies and induce antiviral states in infected cells, potentially preventing cancer progression by targeting viral replication and promoting immune surveillance (62). Conversely, mouse dendritic cells deficient in regulating TBK1-IRF3-dependent type I IFN production are more susceptible to lethal pathogens (63). In addition to antigen-presenting cells, the TBK1-IFN pathway activates innate immune signaling pathways in various immune cells, thereby bolstering the efficacy of tumor immunotherapy. TREX1 inhibits cancer cells from enhancing STING-IFN signaling, which attracts T cells and NK cells, making tumors sensitive to NK cell-derived IFNγ (64). M6A modification promotes the m6A demethylase ALKBH5 in head and neck squamous cell carcinoma to play a key role in promoting the malignant biological behavior of the tumor. RIG-I, as a downstream target of m6A modification, is affected by the overexpression of ALKBH5, which can reduce the killing effect of immune cells by inhibiting the RIG-I mediated TBK1-IRF3-IFN-α pathway (41). Overall, the TBK1-IFN pathway enhances immune cell activation and tumor cell apoptosis, thereby boosting tumor immune response.

### 3.2 Tumor-promoting effect

The activation of the TBK1-IFN-I pathway has been attributed positive significance in anti-tumor responses and improved prognosis in many tumors. However, the complexity of the tumor microenvironment and tumor heterogeneity complicates the effects of IFN-I on malignant tumor proliferation. Certain cancers acquire mutations in the IFN signaling pathway, rendering them resistant to the growth-inhibitory effects of type I IFNs, despite their robust induction of cell cycle arrest (65). Additionally, it is noteworthy that IRF-7, a transcription factor of a crucial interferon-inducing gene in the TBK1-IFN pathway, has demonstrated anti-tumor effects in some studies, while others suggest the possibility of its pro-cancer effects (66).

#### 3.2.1 Induced immune cell exhaustion

T cells play a pivotal role in orchestrating antitumor immune responses and eradicating tumors. In most cases, CD8 T cells serve as the ultimate executors of tumor control, even when other components of the immune system are augmented to combat cancer. Direct IFN-I signaling is crucial for the activation, proliferation, differentiation, and survival of antigen-activated CD8 T cells (67). However, continuous stimulation by type I IFN promotes CD8 T cell depletion (68). Recent findings indicate that TCF19, highly expressed in microsatellite unstable (MSI) endometrial carcinoma compared to microsatellite stabilized (MSS) tumors, plays a dual role in tumor progression. On one hand, it promotes tumor progression through non-immune mechanisms, while on the other hand, it leads to CD8 T cell functional depletion by up-regulating TRIM14 to continuously activate the TBK1-IFNβ pathway. Blocking IFN-β signaling mitigated progressive CD8 T cell dysfunction (46). Furthermore, combined inhibitory therapy targeting TCF19 and PD-1 has been shown to restore CD8 T cell functionality and regain tumor control (46). Similar results were reported by Zeng et al. (69). These findings further substantiate the detrimental impact of TBK1-IFN pathway hyperactivation on immune cell function within tumors.

#### 3.2.2 Induces tumor-related inflammation

The data indicate that dysregulation of the expression of multiple signaling cascade members that regulate IFN production, including TBK1, increases susceptibility to colorectal cancer (CRC) (70). The TBK1-IRF3 pathway acts as a common downstream pathway for the three signaling pathways (RIG-I, TLR, and cGAS-STING) that induce type I IFN transcription. Moreover, this pathway contributes to immune imbalance and inflammatory responses, thereby creating favorable conditions for the progression of inflammatory bowel disease (IBD) to colon cancer. Selective inhibition of upstream signaling in this pathway has emerged as an important approach for ameliorating colitis (71). In addition, key mutations in upstream signaling, such as mutant RIG-I, have been associated with susceptibility to colitis-related colon cancer. Mechanistically, mutant RIG-I directly interacts with DDX3, generating abnormal circular RNA and establishing a non-specific inflammatory stimulation environment conducive to cancer development through the MAVS-TBK1-IRF3-IFN-I signaling cascade (38).

Autophagy regulates the activation of the TBK1-IFN pathway, facilitating crosstalk between metabolism and innate immunity. The regulation of IFN by DNA stimulation depends on autophagy-mediated degradation of STING, a process mediated by TBK1 and IRF3. This degradation is a critical step in preventing excessive inflammation (72). The progression of non-alcoholic steatohepatitis (NASH) to hepatocellular carcinoma (HCC) is well-documented, with chronic inflammation involving type I interferons being a significant contributing factor. Hepatic nuclear factor-1 α (HNF1A), acting as an autophagy cargo receptor, negatively regulates type I IFN by inducing autophagic degradation of TBK1 (73). Studies have shown that downregulation of HNF1A in NASH patients reverses this process, leading to overexpression of type I IFN as TBK1 transitions from a relatively quiescent state to an active state (74).

In the context of the pre-metastatic niche (PMN), we introduce the concept of interaction between *in situ* tumors and distant metastatic tumors at the tumor microenvironment level. The TBK1-IFN-β pathway supports PMN formation, and pharmacological inhibition of this pathway proves to be an effective strategy in preventing melanin lung metastasis (75). Thus, the TBK1-IFN pathway not only promotes anti-tumor immune surveillance but also mediates tumor development through sustained inflammatory stimulation. Targeted therapy for this pathway must achieve a delicate balance between these dual roles.

## 4 Potential of the TBK1-IFN-I pathway in antitumor therapy

### 4.1 Potential in tumor immunotherapy

Signaling of IFN production plays a crucial role in the innate immune pathway, mediating the response of chemotherapy and radiotherapy to PD-L1 induced by DNA damage in tumor cells (76). Tumor cells exposed to IFN stimulate the production of PD-L1 and PD-L2, thereby promoting tumor adaptive resistance (77). This provides a theoretical basis for developing an applied strategy of PD-L1 blocking therapy combined with the stimulation of interferon production in clinical therapy (78).

Activation of the TBK1-IFN pathway may also offer an opportunity for clearing resistant residual tumor cells through immunotherapy. Studies have demonstrated that TBK1-IFN signaling can be enhanced by pemetrexed to improve the immunogenicity of EGFR-TKI resistant non-small cell lung cancer (NSCLC) cells with MET amplification (23). Activated type I IFN induces the production of pro-inflammatory chemokines, which recruit CD8 toxic T cell infiltration, achieved through the inhibition of WEE1 induced by DNA damage (29). Surprisingly, simultaneous activation of the STAT1-IFN-γ pathway also enhances PD-L1 expression (29). Inhibition of the non-autophagy function of FIP200 allows breast cancer to benefit from immune checkpoint inhibitors, also via activation of the TBK1-IRF pathway (79). Improving the therapeutic efficacy of Attilizumab in breast cancer by activating the interferon gene STING upstream of TBK1 appeared to provide strong evidence for this conclusion. However, the negative correlation between PD-L1 and STING expression led the researchers to attribute this result to interferon-mediated inflammatory properties (80). This study did not reassess PD-L1 expression in tumor tissues after STING agonists were used, leaving the question of whether IFN enhances the immunotherapy efficacy of Attilizumab by promoting PD-L1 expression unanswered. It has been reported that EYA2 can inhibit the STING-TBK1-IFN-β pathway by targeting miR-93, thereby promoting uncontrolled growth of breast cancer tumors. Knockout of EYA2 led to a reversal in the expression of IFN-β, ISG, and PD-L1 (81). However, another study confirmed an association between the TBK1-IFN pathway and PD-L1 expression. Researchers found that IL6 stimulated the STING-TBK1-IFN-I pathway by increasing the massive release of mtDNA in EC cells. They observed up-regulation of PD-L1, a downstream gene of IFN, in EC cells, which further inhibited CD3+/CD8+ T cell activity when mtDNA was encapsulated in EV (69). As a downstream gene of TBK1, NF-κB has a binding site near the PD-L1 promoter, which can be activated through TBK1-mediated signaling. This activation further confirms the close molecular association between TBK1 and PD-L1 (45). Notably, in promoting tumor PD-L1 expression, TBK1 and IFN mutually extend and complement each other (28).

These findings indicate that TBK1, as a pivotal node in numerous pathways, interacts with complex and variable upstream signaling molecules that trigger downstream IFN expression. Therefore, focusing on the downstream common TBK1-IFN pathway of multiple signaling pathways is crucial for promoting the efficacy of tumor PD-L1 checkpoint inhibitors. EGFR mutant NSCLC is not typically sensitive to immunotherapy; however, it has been suggested that the use of EGFR inhibitors can up-regulate the expression of PD-L1 through interferon-dependent pathways, enabling these tumors to benefit from immunotherapy (82).

The use of viral infection to stimulate non-malignant components of the tumor microenvironment, especially immune cells represented by macrophages, further triggers downstream anti-tumor immune activity by inducing innate inflammation in local areas. For example, TBK1-IRF3 mediates poliovirus-induced overexpression of type I/III IFN, thereby promoting the antitumor effect of T cells (83). The TBK1-IRF3 signaling pathway serves as a crucial node connecting innate immunity and reversing tumor suppressive immunity, providing an important direction for understanding how to fully stimulate anti-tumor immunity of type I IFN in specific situations.

Pathogens evade clearance by the host immune system through inhibition of autophagy, while host cells appear to have evolved mechanisms to maintain homeostasis by activating the TBK1-IFN pathway through inhibition of selective autophagy. It has been reported that autophagy inhibitors promote the expression of pro-inflammatory factors by activating the TBK1-IFN pathway, thereby enhancing the response of breast cancer to immune checkpoint inhibitors (ICIs) (84). By inhibiting RB1CC1, AZI2 accumulates, leading to overactivation of TBK1-IFN, promotion of cytokine expression in breast cancer, CD8T cell infiltration, and improvement of the response of breast cancer to ICI (84). Interferon-resistant cancer cells, while gaining a growth/survival advantage over normal cells, may have compromised their ability to mount an antiviral response (65). By mimicking viral activation of the TBK1-IFN pathway, “cold” tumors can be “heated,” and the response to ICI can be improved by activating TBK1 splicer proteins STING and MAVS. Recent studies have shown that targeting TBK1 can reduce the sensitivity of tumor cells to effector cytokines (such as TNF-α and IFN-γ), promote tumor cell apoptosis, and reverse tumor resistance to immunotherapy (85).

### 4.2 Potential applications in other therapies

Gamma irradiated colorectal cancer cell lines have been shown to induce IFNL1 (a type III interferon) production via the TBK1-IRF1 pathway. Additionally, IFNL1 enhances its own expression through the upregulation of positive kinase feedback within this signaling cascade (86). Sorafenib, commonly used as a chemotherapeutic agent for HCC treatment, relies on autophagy-mediated degradation of key components in the MAVs-STING-TBK1-IFN-I pathway and the inhibition of type I IFN production by modulating the interaction between IRF3 and splicing proteins. Despite these side effects, which diminish antitumor efficacy, contradicting its primary purpose as an anticancer drug, this very inhibition of the TBK1-IFN-mediated innate immune pathway expands sorafenib’s potential in managing HCC recurrence post-liver transplantation (73). Activation of the TBK1-IFN pathway by MEDI2228 enhances CD38 expression in multiple myeloma cells, thereby augmenting the anti-tumor effects of CD38-targeting antibody-drug conjugates (87). Tiopanib, a pan-PARP inhibitor, exploits the therapeutic vulnerability of PARPi-resistant tumor cells by activating the TBK1-IFN pathway. Even in cells with homologous recombination defects, a common PARPi resistance mechanism, Tiopanib demonstrates high antitumor activity, improving response rates of BRCA-deficient tumors to PARPi. Its mechanism underscores the interplay between innate immunity and anti-tumor immunity (88). Notably, activation of the TBK1-IRF3-IFN-I pathway has been identified as an independent mechanism of adaptive resistance induced by EGFR TKI in NSCLCs with EGFR mutations. The combination of EGFR inhibition and type I interferon inhibitors can enhance the effectiveness against EGFR-mutant cells and overcome primary resistance in EGFR wild type NSCLC (27).

A comprehensive understanding of the TBK1-IFN pathway may offer insights into the restricted clinical utility of TBK1 inhibitors and present novel perspectives for their development and utilization.

## 5 Conclusion

TBK1, which is expressed in almost all tissues, has complex upstream signals related to its activation and mediates downstream pathways that span multiple stages of tumor development. It participates in various physiological processes of tumors, including uncontrolled growth (31), immune evasion (28), tumor metabolism (89), and the creation of a tumor inflammatory environment (38). KRAS mutations are widespread in a variety of aggressive tumors, but there is a lack of effective targeted therapies. However, in a systematic RNA interference experiment, the synthetic lethality of TBK1 and KRAS was confirmed (14). TBK1 induces anti-apoptosis in KRAS-mutated non-small cell lung cancer cell lines by activating NF-κB, and the TBK1-mediated pathway has been further investigated in various other KRAS-mutated tumors (39). Nonetheless, the effectiveness of TBK1 inhibitors for tumor suppression is not universally applicable (16). Firstly, the efficacy and specificity of the drug itself must be considered. Most TBK1 inhibitors have inhibitory effects on IKKϵ (49), a non-classical member of the IκB kinase family. Although IKKϵ can provide compensatory expression when TBK1 is inhibited (90), its role in tumors cannot be ignored (91), and there is still a lack of small molecule TBK1 selective inhibitors for tumors. Secondly, tumors sensitive to TBK1 inhibition are influenced by genetic and epigenetic factors. For example, the effect of TBK1 inhibitors in KRAS mutant NSCLC cell lines containing TP53 and LKB1 co-mutations is influenced by the state of the transcriptional cells (33).

Moreover, even in most tumors, TBK1 still has the tag of immune escape genes, but it is undeniable that it still has the effect of activating innate immunity at the early stage of tumor development, and its importance cannot be ignored. Therefore, this review synthesized the signaling pathways and physiological effects mediated by TBK1 in different tumor backgrounds to visually demonstrate the duplex nature of TBK1 in tumor development, in order to provide a reference for the rational application of TBK1 inhibitors in tumors.

Based on this, we further found that the TBK1-IFN pathway, which is generally believed to mediate the anti-tumor effect of innate immunity, also has a pro-tumor effect. In particular, in the early stages of tumor development, the TBK1-IFN pathway creates a local inflammatory environment by stimulating immune cell activation and anti-tumor cytokine secretion. However, as inflammation persists, the TBK1-IFN pathway leads to the depletion of immune cells and the creation of an inflammatory microenvironment suitable for tumor growth and distant metastasis colonization, which is particularly evident during the development of intestinal tumors.

Furthermore, STING agonists are a direct and effective means to promote the expression of the TBK1-IFN pathway, and an in-depth understanding of TBK1-IFN is conducive to the effective application of STING agonists. Common clinical treatments such as chemoradiotherapy can stimulate the TBK1-IFN pathway to inhibit tumor growth, but it is also necessary to avoid inhibiting the TBK1-IFN pathway that plays an anti-tumor role, such as sorafenib. It has also been found that stimulating IFN through the metabolic-immune pathway appears to mitigate the pro-tumor effects associated with long-term activation of IFN (92).

It is worth noting that TBK1-IFN can indirectly promote the expression of PD-L1 in tumors by activating T cells and NK cells to secrete IFNγ, which may further guide the use of immunosuppressants. Special attention should be given to the modulation of the tumor microenvironment by TBK1-IFN, as targeting or activating this pathway may offer strategies to enhance the efficacy of immunotherapy. The anti-tumor and pro-tumor effects of TBK1-IFN are dynamic processes, and the appropriate time to target this pathway still needs to be further explored. Thus, the evaluation of immunosuppressive status in the tumor microenvironment may be an important clue.

In conclusion, TBK1 exhibits dual roles in tumor progression, with the expression of the IFN pathway potentially offering a crucial explanation for this contradiction. Exploring interventions targeting the TBK1-IFN pathway in specific tumor contexts is imperative for enhancing therapeutic efficacy in cancer treatment. The untapped potential of this pathway in augmenting the effectiveness of immunotherapy warrants further development and investigation.

## Author contributions

BW: Writing – original draft, Writing – review & editing. FZ: Conceptualization, Software, Data curation, Formal analysis, Funding acquisition, Investigation, Methodology, Project administration, Resources, Supervision, Validation, Visualization, Writing – review & editing. XW: Data curation, Methodology, Conceptualization, Formal analysis, Funding acquisition, Investigation, Project administration, Resources, Software, Supervision, Validation, Visualization, Writing – review & editing. MJ: Conceptualization, Funding acquisition, Project administration, Supervision, Visualization, Data curation, Formal analysis, Investigation, Methodology, Resources, Software, Validation, Writing – review & editing.

## References

[1] Shen RR Hahn WC. Emerging roles for the non-canonical IKKs in cancer. Oncogene. (2011) 30:631–41. doi: 10.1038/onc.2010.493 PMC3235643 21042276

[2] Sharma S tenOever BR Grandvaux N Zhou GP Lin R Hiscott J. Triggering the interferon antiviral response through an IKK-related pathway. Science. (2003) 300:1148–51. doi: 10.1126/science.1081315 12702806

[3] Hasan M Yan N. Therapeutic potential of targeting TBK1 in autoimmune diseases and interferonopathies. Pharmacol Res. (2016) 111:336–42. doi: 10.1016/j.phrs.2016.04.008 PMC5703047 27353409

[4] Korherr C Gille H Schäfer R Koenig-Hoffmann K Dixelius J Egland KA. Identification of proangiogenic genes and pathways by high-throughput functional genomics: TBK1 and the IRF3 pathway. Proc Natl Acad Sci USA. (2006) 103:4240–5. doi: 10.1073/pnas.0511319103 PMC1449677 16537515

[5] Herhaus L. TBK1 (TANK-binding kinase 1)-mediated regulation of autophagy in health and disease. Matrix Biol. (2021) 100-101:84–98. doi: 10.1016/j.matbio.2021.01.004 33454423

[6] Kim JY Welsh EA Oguz U Fang B Bai Y Kinose F. Dissection of TBK1 signaling via phosphoproteomics in lung cancer cells. Proc Natl Acad Sci USA. (2013) 110:12414–9. doi: 10.1073/pnas.1220674110 PMC3725062 23836654

[7] Pillai S Nguyen J Johnson J Haura E Coppola D Chellappan S. Tank binding kinase 1 is a centrosome-associated kinase necessary for microtubule dynamics and mitosis. Nat Commun. (2015) 6:10072. doi: 10.1038/ncomms10072 26656453 PMC4682058

[8] Zhang Y Unnithan RVM Hamidi A Caja L Saupe F Moustakas A. TANK-binding kinase 1 is a mediator of platelet-induced EMT in mammary carcinoma cells. FASEB J. (2019) 33:7822–32. doi: 10.1096/fj.201801936RRR 30912981

[9] Lukhele S Boukhaled GM Brooks DG. Type I interferon signaling, regulation and gene stimulation in chronic virus infection. Semin Immunol. (2019) 43:101277. doi: 10.1016/j.smim.2019.05.001 31155227 PMC8029807

[10] Boukhaled GM Harding S Brooks DG. Opposing roles of type I interferons in cancer immunity. Annu Rev Pathol. (2021) 16:167–98. doi: 10.1146/annurev-pathol-031920-093932 PMC8063563 33264572

[11] Dhanwani R Takahashi M Sharma S. Cytosolic sensing of immuno-stimulatory DNA, the enemy within. Curr Opin Immunol. (2018) 50:82–7. doi: 10.1016/j.coi.2017.11.004 PMC5916810 29247853

[12] Ma H Yang W Zhang L Liu S Zhao M Zhou G. Interferon-alpha promotes immunosuppression through IFNAR1/STAT1 signalling in head and neck squamous cell carcinoma. Br J Cancer. (2019) 120:317–30. doi: 10.1038/s41416-018-0352-y PMC6353953 30555157

[13] Kiessling MK Schuierer S Stertz S Beibel M Bergling S Knehr J. et al: Identification of oncogenic driver mutations by genome-wide CRISPR-Cas9 dropout screening. BMC Genomics. (2016) 17:723. doi: 10.1186/s12864-016-3042-2 27613601 PMC5016932

[14] Barbie DA Tamayo P Boehm JS Kim SY Moody SE Dunn IF. et al: Systematic RNA interference reveals that oncogenic KRAS-driven cancers require TBK1. Nature. (2009) 462:108–12. doi: 10.1038/nature08460 PMC2783335 19847166

[15] Muvaffak A Pan Q Yan H Fernandez R Lim J Dolinski B. Evaluating TBK1 as a therapeutic target in cancers with activated IRF3. Mol Cancer Res. (2014) 12:1055–66. doi: 10.1158/1541-7786.MCR-13-0642 24752990

[16] Revach OY Liu S Jenkins RW. Targeting TANK-binding kinase 1 (TBK1) in cancer. Expert Opin Ther Targets. (2020) 24:1065–78. doi: 10.1080/14728222.2020.1826929 PMC7644630 32962465

[17] Liang J Hong Z Sun B Guo Z Wang C Zhu J. The alternatively spliced isoforms of key molecules in the cGAS-STING signaling pathway. Front Immunol. (2021) 12:771744. doi: 10.3389/fimmu.2021.771744 34868032 PMC8636596

[18] Helgason E Phung QT Dueber EC. Recent insights into the complexity of Tank-binding kinase 1 signaling networks: the emerging role of cellular localization in the activation and substrate specificity of TBK1. FEBS Lett. (2013) 587:1230–7. doi: 10.1016/j.febslet.2013.01.059 23395801

[19] Zhu L Li Y Xie X Zhou X Gu M Jie Z. TBKBP1 and TBK1 form a growth factor signalling axis mediating immunosuppression and tumourigenesis. Nat Cell Biol. (2019) 21:1604–14. doi: 10.1038/s41556-019-0429-8 PMC6901116 31792381

[20] Shi JH Xie X Sun SC. TBK1 as a regulator of autoimmunity and antitumor immunity. Cell Mol Immunol. (2018) 15:743–5. doi: 10.1038/cmi.2017.165 PMC6141483 29503440

[21] An X Zhu Y Zheng T Wang G Zhang M Li J. et al: An Analysis of the Expression and Association with Immune Cell Infiltration of the cGAS/STING Pathway in Pan-Cancer. Mol Ther Nucleic Acids. (2019) 14:80–9. doi: 10.1016/j.omtn.2018.11.003 PMC6305687 30583098

[22] Zhao X Cao Y Lu R Zhou Z Huang C Li L. et al: Phosphorylation of AGO2 by TBK1 Promotes the Formation of Oncogenic miRISC in NSCLC. Adv Sci (Weinh). (2024) 11:e2305541. doi: 10.1002/advs.202305541 38351659 PMC11022703

[23] Yoshida R Saigi M Tani T Springer BF Shibata H Kitajima S. MET-induced CD73 restrains STING-mediated immunogenicity of EGFR-mutant lung cancer. Cancer Res. (2022) 82:4079–92. doi: 10.1158/0008-5472.CAN-22-0770 PMC9627131 36066413

[24] Woo SR Fuertes MB Corrales L Spranger S Furdyna MJ Leung MY. STING-dependent cytosolic DNA sensing mediates innate immune recognition of immunogenic tumors. Immunity. (2014) 41:830–42. doi: 10.1016/j.immuni.2014.10.017 PMC4384884 25517615

[25] Zhu Y An X Zhang X Qiao Y Zheng T Li X. STING: a master regulator in the cancer-immunity cycle. Mol Cancer. (2019) 18:152. doi: 10.1186/s12943-019-1087-y 31679519 PMC6827255

[26] Yang JY Jie Z Mathews A Zhou X Li Y Gu M. Intestinal epithelial TBK1 prevents differentiation of T-helper 17 cells and tumorigenesis in mice. Gastroenterology. (2020) 159:1793–806. doi: 10.1053/j.gastro.2020.07.047 PMC7680348 32745468

[27] Gong K Guo G Panchani N Bender ME Gerber DE Minna JD. EGFR inhibition triggers an adaptive response by co-opting antiviral signaling pathways in lung cancer. Nat Cancer. (2020) 1:394–409. doi: 10.1038/s43018-020-0048-0 33269343 PMC7706867

[28] Breitenecker K Homolya M Luca AC Lang V Trenk C Petroczi G. Down-regulation of A20 promotes immune escape of lung adenocarcinomas. Sci Transl Med. (2021) 13. doi: 10.1126/scitranslmed.abc3911 PMC7611502 34233950

[29] Taniguchi H Caeser R Chavan SS Zhan YA Chow A Manoj P. WEE1 inhibition enhances the antitumor immune response to PD-L1 blockade by the concomitant activation of STING and STAT1 pathways in SCLC. Cell Rep. (2022) 39:110814. doi: 10.1016/j.celrep.2022.110814 35584676 PMC9449677

[30] Newman AC Scholefield CL Kemp AJ Newman M McIver EG Kamal A. TBK1 kinase addiction in lung cancer cells is mediated via autophagy of Tax1bp1/Ndp52 and non-canonical NF-κB signalling. PloS One. (2012) 7:e50672. doi: 10.1371/journal.pone.0050672 23209807 PMC3510188

[31] Cooper JM Ou YH McMillan EA Vaden RM Zaman A Bodemann BO. TBK1 provides context-selective support of the activated AKT/mTOR pathway in lung cancer. Cancer Res. (2017) 77:5077–94. doi: 10.1158/0008-5472.CAN-17-0829 PMC5833933 28716898

[32] Liu W Huang YJ Liu C Yang YY Liu H Cui JG. Inhibition of TBK1 attenuates radiation-induced epithelial-mesenchymal transition of A549 human lung cancer cells via activation of GSK-3β and repression of ZEB1. Lab Invest. (2014) 94:362–70. doi: 10.1038/labinvest.2013.153 24468793

[33] Kitajima S Ivanova E Guo S Yoshida R Campisi M Sundararaman SK. Suppression of STING associated with LKB1 loss in KRAS-Driven lung cancer. Cancer Discov. (2019) 9:34–45. doi: 10.1158/2159-8290.CD-18-0689 30297358 PMC6328329

[34] Parkes EE Walker SM Taggart LE McCabe N Knight LA Wilkinson R. Activation of STING-Dependent innate immune signaling by S-Phase-Specific DNA damage in breast cancer. J Natl Cancer Inst. (2017) 109. doi: 10.1093/jnci/djw199 PMC5441301 27707838

[35] Yang KM Jung Y Lee JM Kim W Cho JK Jeong J. Loss of TBK1 induces epithelial-mesenchymal transition in the breast cancer cells by ERα downregulation. Cancer Res. (2013) 73:6679–89. doi: 10.1158/0008-5472.CAN-13-0891 24062311

[36] Linares JF Zhang X Martinez-Ordoñez A Duran A Kinoshita H Kasashima H. PKCλ/ι inhibition activates an ULK2-mediated interferon response to repress tumorigenesis. Mol Cell. (2021) 81:4509–4526.e4510. doi: 10.1016/j.molcel.2021.08.039 34560002 PMC8571054

[37] Ritter JL Zhu Z Thai TC Mahadevan NR Mertins P Knelson EH. Phosphorylation of RAB7 by TBK1/IKKϵ Regulates innate immune signaling in triple-Negative breast cancer. Cancer Res. (2020) 80:44–56. doi: 10.1158/0008-5472.CAN-19-1310 31662325 PMC6942622

[38] Song J Zhao W Zhang X Tian W Zhao X Ma L. Mutant RIG-I enhances cancer-related inflammation through activation of circRIG-I signaling. Nat Commun. (2022) 13:7096. doi: 10.1038/s41467-022-34885-3 36402769 PMC9675819

[39] Cruz VH Arner EN Du W Bremauntz AE Brekken RA. Axl-mediated activation of TBK1 drives epithelial plasticity in pancreatic cancer. JCI Insight. (2019) 5. doi: 10.1101/450049 PMC6538328 30938713

[40] Hu L Xie H Liu X Potjewyd F James LI Wilkerson EM. TBK1 is a synthetic lethal target in cancer with VHL loss. Cancer Discov. (2020) 10:460–75. doi: 10.1158/2159-8290.CD-19-0837 PMC7058506 31810986

[41] Jin S Li M Chang H Wang R Zhang Z Zhang J. The m6A demethylase ALKBH5 promotes tumor progression by inhibiting RIG-I expression and interferon alpha production through the IKKϵ/TBK1/IRF3 pathway in head and neck squamous cell carcinoma. Mol Cancer. (2022) 21:97. doi: 10.1186/s12943-022-01572-2 35395767 PMC8994291

[42] Alvarado AG Thiagarajan PS Mulkearns-Hubert EE Silver DJ Hale JS Alban TJ. Glioblastoma cancer stem cells evade innate immune suppression of self-Renewal through reduced TLR4 expression. Cell Stem Cell. (2017) 20:450–461.e454. doi: 10.1016/j.stem.2016.12.001 28089910 PMC5822422

[43] Zhang J Chen Y Chen X Zhang W Zhao L Weng L. Deubiquitinase USP35 restrains STING-mediated interferon signaling in ovarian cancer. Cell Death Differ. (2021) 28:139–55. doi: 10.1038/s41418-020-0588-y PMC7853139 32678307

[44] Bruand M Barras D Mina M Ghisoni E Morotti M Lanitis E. Cell-autonomous inflammation of BRCA1-deficient ovarian cancers drives both tumor-intrinsic immunoreactivity and immune resistance via STING. Cell Rep. (2021) 36:109412. doi: 10.1016/j.celrep.2021.109412 34289354 PMC8371260

[45] Cai H Yan L Liu N Xu M Cai H. IFI16 promotes cervical cancer progression by upregulating PD-L1 in immunomicroenvironment through STING-TBK1-NF-kB pathway. BioMed Pharmacother. (2020) 123:109790. doi: 10.1016/j.biopha.2019.109790 31896065

[46] Ma X Wang Q Sun C Agarwal I Wu H Chen J. Targeting TCF19 sensitizes MSI endometrial cancer to anti-PD-1 therapy by alleviating CD8(+) T cell exhaustion via TRIM14-IFN-β axis. Cell Rep. (2023) 42:112944. doi: 10.1016/j.celrep.2023.112944 37566545

[47] Cheng C Ji Z Sheng Y Wang J Sun Y Zhao H. Aphthous ulcer drug inhibits prostate tumor metastasis by targeting IKKε/TBK1/NF-κB signaling. Theranostics. (2018) 8:4633–48. doi: 10.7150/thno.26687 PMC6160770 30279728

[48] Meng F Yu Z Zhang D Chen S Guan H Zhou R. Induced phase separation of mutant NF2 imprisons the cGAS-STING machinery to abrogate antitumor immunity. Mol Cell. (2021) 81:4147–4164.e4147. doi: 10.1016/j.molcel.2021.07.040 34453890

[49] Liu S Marneth AE Alexe G Walker SR Gandler HI Ye DQ. The kinases IKBKE and TBK1 regulate MYC-dependent survival pathways through YB-1 in AML and are targets for therapy. Blood Adv. (2018) 2:3428–42. doi: 10.1182/bloodadvances.2018016733 PMC6290107 30504235

[50] Vu HL Aplin AE. Targeting TBK1 inhibits migration and resistance to MEK inhibitors in mutant NRAS melanoma. Mol Cancer Res. (2014) 12:1509–19. doi: 10.1158/1541-7786.MCR-14-0204 PMC4482471 24962318

[51] Seguin L Kato S Franovic A Camargo MF Lesperance J Elliott KC. An integrin β₃-KRAS-RalB complex drives tumour stemness and resistance to EGFR inhibition. Nat Cell Biol. (2014) 16:457–68. doi: 10.1038/ncb2953 PMC4105198 24747441

[52] Goubau D Deddouche S Reis e Sousa C. Cytosolic sensing of viruses. Immunity. (2013) 38:855–69. doi: 10.1016/j.immuni.2013.05.007 PMC7111113 23706667

[53] Guo B Cheng G. Modulation of the interferon antiviral response by the TBK1/IKKi adaptor protein TANK. J Biol Chem. (2007) 282:11817–26. doi: 10.1074/jbc.M700017200 17327220

[54] Fujita F Taniguchi Y Kato T Narita Y Furuya A Ogawa T. Identification of NAP1, a regulatory subunit of IkappaB kinase-related kinases that potentiates NF-kappaB signaling. Mol Cell Biol. (2003) 23:7780–93. doi: 10.1128/MCB.23.21.7780-7793.2003 PMC207563 14560022

[55] Ryzhakov G Randow F. SINTBAD, a novel component of innate antiviral immunity, shares a TBK1-binding domain with NAP1 and TANK. EMBO J. (2007) 26:3180–90. doi: 10.1038/sj.emboj.7601743 PMC1914091 17568778

[56] Fitzgerald KA McWhirter SM Faia KL Rowe DC Latz E Golenbock DT. IKKepsilon and TBK1 are essential components of the IRF3 signaling pathway. Nat Immunol. (2003) 4:491–6. doi: 10.1038/ni921 12692549

[57] Xia P Wang S Gao P Gao G Fan Z. DNA sensor cGAS-mediated immune recognition. Protein Cell. (2016) 7:777–91. doi: 10.1007/s13238-016-0320-3 PMC5084157 27696330

[58] Agalioti T Lomvardas S Parekh B Yie J Maniatis T Thanos D. Ordered recruitment of chromatin modifying and general transcription factors to the IFN-beta promoter. Cell. (2000) 103:667–78. doi: 10.1016/S0092-8674(00)00169-0 11106736

[59] Ma Z Jacobs SR West JA Stopford C Zhang Z Davis Z. Modulation of the cGAS-STING DNA sensing pathway by gammaherpesviruses. Proc Natl Acad Sci USA. (2015) 112:E4306–4315. doi: 10.1073/pnas.1503831112 PMC4534226 26199418

[60] Zhu M Tang X Zhu Z Gong Z Tang W Hu Y. STING activation in macrophages by vanillic acid exhibits antineoplastic potential. Biochem Pharmacol. (2023) 213:115618. doi: 10.1016/j.bcp.2023.115618 37211172

[61] Zou Z Li M Zhou Y Li J Pan T Lai L. Tumor necrosis factor-α-induced protein 8-like 2 negatively regulates innate immunity against RNA virus by targeting RIG-I in macrophages. Front Immunol. (2021) 12:642715. doi: 10.3389/fimmu.2021.642715 33815396 PMC8017232

[62] Stanley MA Pett MR Coleman N. HPV: from infection to cancer. Biochem Soc Trans. (2007) 35:1456–60. doi: 10.1042/BST0351456 18031245

[63] Liu Z Jiang C Lei Z Dong S Kuang L Huang C. Phospholipase A2 inhibitor and LY6/PLAUR domain-containing protein PINLYP regulates type I interferon innate immunity. Proc Natl Acad Sci USA. (2022) 119. doi: 10.1073/pnas.2111115119 PMC8740751 34969857

[64] Tani T Mathsyaraja H Campisi M Li ZH Haratani K Fahey CG. TREX1 inactivation unleashes cancer cell STING-interferon signaling and promotes antitumor immunity. Cancer Discov. (2024) 14:752–65. doi: 10.1158/2159-8290.CD-23-0700 PMC11062818 38227896

[65] Stojdl DF Lichty B Knowles S Marius R Atkins H Sonenberg N. Exploiting tumor-specific defects in the interferon pathway with a previously unknown oncolytic virus. Nat Med. (2000) 6:821–5. doi: 10.1038/77558 10888934

[66] Zhang L Zhang J Lambert Q Der CJ Del Valle L Miklossy J. Interferon regulatory factor 7 is associated with Epstein-Barr virus-transformed central nervous system lymphoma and has oncogenic properties. J Virol. (2004) 78:12987–95. doi: 10.1128/JVI.78.23.12987-12995.2004 PMC524977 15542650

[67] Katlinski KV Gui J Katlinskaya YV Ortiz A Chakraborty R Bhattacharya S. Inactivation of interferon receptor promotes the establishment of immune privileged tumor microenvironment. Cancer Cell. (2017) 31:194–207. doi: 10.1016/j.ccell.2017.01.004 28196594 PMC5313042

[68] Wherry EJ Kurachi M. Molecular and cellular insights into T cell exhaustion. Nat Rev Immunol. (2015) 15:486–99. doi: 10.1038/nri3862 PMC4889009 26205583

[69] Zeng X Li X Zhang Y Cao C Zhou Q. IL6 Induces mtDNA Leakage to Affect the Immune Escape of Endometrial Carcinoma via cGAS-STING. J Immunol Res. (2022) 2022:3815853. doi: 10.1155/2022/3815853 35692503 PMC9184159

[70] Catalano C da Silva Filho MI Frank C Lu S Jiraskova K Vymetalkova V. Epistatic effect of TLR3 and cGAS-STING-IKKϵ-TBK1-IFN signaling variants on colorectal cancer risk. Cancer Med. (2020) 9:1473–84. doi: 10.1002/cam4.2804 PMC7013077 31869529

[71] Cai Y Xu B Zhou F Wu J Li S Zheng Q. Si-Ni-San ameliorates chronic colitis by modulating type I interferons-mediated inflammation. Phytomedicine. (2021) 84:153495. doi: 10.1016/j.phymed.2021.153495 33611210

[72] Prabakaran T Bodda C Krapp C Zhang BC Christensen MH Sun C. Attenuation of cGAS-STING signaling is mediated by a p62/SQSTM1-dependent autophagy pathway activated by TBK1. EMBO J. (2018) 37. doi: 10.15252/embj.201797858 PMC5897779 29496741

[73] Huang Y Liang W Li K Liao X Chen J Qiu X. Sorafenib suppresses the activation of type I interferon pathway induced by RLR-MAVS and cGAS-STING signaling. Biochem Biophys Res Commun. (2022) 623:181–8. doi: 10.1016/j.bbrc.2022.07.028 35921710

[74] He J Du C Peng X Hong W Qiu D Qiu X. Hepatocyte nuclear factor 1A suppresses innate immune response by inducing degradation of TBK1 to inhibit steatohepatitis. Genes Dis. (2023) 10:1596–612. doi: 10.1016/j.gendis.2022.05.029 PMC10311033 37397525

[75] Tu S Mao D Shi M Zhang H Liu C Li X. Icaritin ameliorates extracellular microparticles-induced inflammatory pre-metastatic niche via modulating the cGAS-STING signaling. Phytother Res. (2022) 36:2127–42. doi: 10.1002/ptr.7433 35257426

[76] Jiang M Jia K Wang L Li W Chen B Liu Y. Alterations of DNA damage response pathway: Biomarker and therapeutic strategy for cancer immunotherapy. Acta Pharm Sin B. (2021) 11:2983–94. doi: 10.1016/j.apsb.2021.01.003 PMC8546664 34729299

[77] Garcia-Diaz A Shin DS Moreno BH Saco J Escuin-Ordinas H Rodriguez GA. Interferon receptor signaling pathways regulating PD-L1 and PD-L2 expression. Cell Rep. (2017) 19:1189–201. doi: 10.1016/j.celrep.2017.04.031 PMC6420824 28494868

[78] Zhou L Xu Q Huang L Jin J Zuo X Zhang Q. et al: Low-dose carboplatin reprograms tumor immune microenvironment through STING signaling pathway and synergizes with PD-1 inhibitors in lung cancer. Cancer Lett. (2021) 500:163–71. doi: 10.1016/j.canlet.2020.11.049 33278498

[79] Okamoto T Yeo SK Hao M Copley MR Haas MA Chen S. FIP200 suppresses immune checkpoint therapy responses in breast cancers by limiting AZI2/TBK1/IRF signaling independent of its canonical autophagy function. Cancer Res. (2020) 80:3580–92. doi: 10.1158/0008-5472.CAN-20-0519 PMC7483892 32580962

[80] Yin M Hu J Yuan Z Luo G Yao J Wang R. STING agonist enhances the efficacy of programmed death-ligand 1 monoclonal antibody in breast cancer immunotherapy by activating the interferon-β signalling pathway. Cell Cycle. (2022) 21:767–79. doi: 10.1080/15384101.2022.2029996 PMC8973354 35130108

[81] Ren L Guo D Wan X Qu R. EYA2 upregulates miR-93 to promote tumorigenesis of breast cancer by targeting and inhibiting the STING signaling pathway. Carcinogenesis. (2022) 43(12):1121–30. doi: 10.1093/carcin/bgab001 33449106

[82] Cheng AN Cheng LC Kuo CL Lo YK Chou HY Chen CH. Mitochondrial Lon-induced mtDNA leakage contributes to PD-L1-mediated immunoescape via STING-IFN signaling and extracellular vesicles. J Immunother Cancer. (2020) 8. doi: 10.1136/jitc-2020-001372 PMC7713199 33268351

[83] Brown MC Mosaheb MM Mohme M McKay ZP Holl EK Kastan JP. Viral infection of cells within the tumor microenvironment mediates antitumor immunotherapy via selective TBK1-IRF3 signaling. Nat Commun. (2021) 12:1858. doi: 10.1038/s41467-021-22088-1 33767151 PMC7994570

[84] Yeo SK Haas M Manupati K Hao M Yang F Chen S. AZI2 mediates TBK1 activation at unresolved selective autophagy cargo receptor complexes with implications for CD8 T-cell infiltration in breast cancer. Autophagy. (2024) 20(3):525–40. doi: 10.1080/15548627.2023.2259775 37733921 PMC10936636

[85] Kelliher MA Fitzgerald KA. TBK1 inhibition unleashes RIPK1, resensitizing tumors to immunotherapy. Trends Immunol. (2023) 44:156–8. doi: 10.1016/j.it.2023.01.009 36740513

[86] Chen J Markelc B Kaeppler J Ogundipe VML Cao Y McKenna WG. STING-dependent interferon-λ1 induction in HT29 cells, a human colorectal cancer cell line, after gamma-radiation. Int J Radiat Oncol Biol Phys. (2018) 101:97–106. doi: 10.1016/j.ijrobp.2018.01.091 29619982

[87] Xing L Wang S Liu J Yu T Chen H Wen K. BCMA-specific ADC MEDI2228 and daratumumab induce synergistic myeloma cytotoxicity via IFN-driven immune responses and enhanced CD38 expression. Clin Cancer Res. (2021) 27:5376–88. doi: 10.1158/1078-0432.CCR-21-1621 PMC8783926 34301753

[88] Wang LM Wang P Chen XM Yang H Song SS Song Z. Thioparib inhibits homologous recombination repair, activates the type I IFN response, and overcomes olaparib resistance. EMBO Mol Med. (2023) 15:e16235. doi: 10.15252/emmm.202216235 36652375 PMC9994488

[89] Zhao P Wong KI Sun X Reilly SM Uhm M Liao Z. TBK1 at the crossroads of inflammation and energy homeostasis in adipose tissue. Cell. (2018) 172:731–743.e712. doi: 10.1016/j.cell.2018.01.007 29425491 PMC5808582

[90] Lafont E Draber P Rieser E Reichert M Kupka S de Miguel D. TBK1 and IKKϵ prevent TNF-induced cell death by RIPK1 phosphorylation. Nat Cell Biol. (2018) 20:1389–99. doi: 10.1038/s41556-018-0229-6 PMC6268100 30420664

[91] Rajurkar M Dang K Fernandez-Barrena MG Liu X Fernandez-Zapico ME Lewis BC. IKBKE is required during KRAS-induced pancreatic tumorigenesis. Cancer Res. (2017) 77:320–9. doi: 10.1158/0008-5472.CAN-15-1684 PMC5243176 28069799

[92] Mu M Niu W Chu F Dong Q Hu S Niu C. CircSOBP suppresses the progression of glioma by disrupting glycolysis and promoting the MDA5-mediated immune response. iScience. (2023) 26:107897. doi: 10.1016/j.isci.2023.107897 37766977 PMC10520879

